# The Risk of Endometrial Cancer and Uterine Sarcoma Following Endometriosis or Pelvic Inflammatory Disease

**DOI:** 10.3390/cancers15030833

**Published:** 2023-01-29

**Authors:** Jing-Yang Huang, Kevin Sheng-Kai Ma, Li-Tzu Wang, Cho-Han Chiang, Shun-Fa Yang, Chun-Hao Wang, Po-Hui Wang

**Affiliations:** 1 Institute of Medicine, Chung Shan Medical University, Taichung 40201, Taiwan; 2 Department of Medical Research, Chung Shan Medical University Hospital, Taichung 40201, Taiwan; 3 Department of Epidemiology, Harvard T.H. Chan School of Public Health, Boston, MA 02115, USA; 4 Center for Global Health, Perelman School of Medicine, University of Pennsylvania, Philadelphia, PA 19104, USA; 5 Department of Dermatology, Massachusetts General Hospital, Boston, MA 02114, USA; 6 Department of Orthodontics and Dentofacial Orthopedics, Henry M. Goldman School of Dental Medicine, Boston University, Boston, MA 02118, USA; 7 Department of Obstetrics & Gynecology, National Taiwan University Hospital & College of Medicine, Taipei 11031, Taiwan; 8 Department of Medicine, Mount Auburn Hospital, Cambridge, MA 02138, USA; 9 Harvard Medical School, Harvard University, Boston, MA 02115, USA; 10 Department of Medicine, National Taiwan University, Taipei 10617, Taiwan; 11 School of Medicine, Chung Shan Medical University, Taichung 40201, Taiwan; 12 Department of Obstetrics and Gynecology, Chung Shan Medical University Hospital, Taichung 40201, Taiwan

**Keywords:** endometriosis, pelvic inflammatory disease, uterine corpus cancer, time varying effect, age subgroups, risk difference, adjusted hazard ratio

## Abstract

**Simple Summary:**

It was revealed in the present population-based cohort study that patients with endometriosis had a significantly increased risk of uterine corpus cancer than did propensity score-matched controls. Moreover, it was found that patients with endometriosis were susceptible to not only endometrial cancer, but also uterine sarcoma. On the contrary, patients with pelvic inflammatory disease did not exhibit an apparent risk of uterine cancer. Overall, endometriosis, but not pelvic inflammatory disease, was an independent risk factor for both endometrial cancer and uterine sarcoma.

**Abstract:**

The relationship between uterine corpus cancer and endometriosis was conflicting. We aimed to determine the risk of uterine cancer in patients with endometriosis or pelvic inflammatory disease (PID). In this population-based cohort study, a total of 135,236 females with endometriosis (*n* = 20,510) or PID (*n* = 114,726), as well as 135,236 age-matched controls, were included. Cox regression models estimated the risk of uterine cancer in each group. Sub-outcomes of risk for uterine corpus cancer included endometrial cancer and uterine sarcoma were analyzed. An age subgroup analysis was performed to determine the moderator effect of age. A landmark analysis depicted the time varying effect of endometriosis and PID. A propensity score matching analysis was conducted to validate the findings. Patients with endometriosis had significantly higher risk of endometrial cancer (adjusted hazard ratio, aHR = 2.92; 95% CI = 2.12–4.03) and uterine sarcoma (aHR = 5.83; 95% CI = 2.02–16.89), while PID was not associated with the risk of uterine cancer. The increased risk of uterine cancer in patients with endometriosis persisted after propensity score matching (aHR = 2.83, 95%CI = 1.70–4.71). The greatest risk of endometrial cancer occurred in patients who had endometriosis for 37 to 60 months (adjusted relative risk, aRR = 9.15, 95% CI = 4.40–19.02). Females aged 12 to 35 years were at the greatest risk of endometriosis-associated uterine cancer (RR = 6.97, 95% CI = 3.41–14.26). In conclusion, patients with endometriosis were at great risk of uterine cancer, including endometrial cancer and uterine sarcoma, compared with propensity score-matched populations and compared with patients of PID. Younger females with endometriosis and patients who had endometriosis for three to five years were at the greatest risk of endometriosis-associated uterine cancer.

## 1. Introduction

Endometriosis is defined as the abnormal implantation of endometrial glands and stroma at ectopic sites even at myometrium as adenomyosis, resulting in chronic inflammation, pain, and infertility [1]. It occurs in approximately 10% of premenopausal women [2]. The incidence rate of endometrial cancer based on the study of Ciou et al. was estimated to be 28.81 per 100,000 endometriosis patients per year, as compared 12.00 per 100,000 non-endometriosis women per year [3]. The crude hazard ratio and adjusted hazard ratio were 2.84 (95% CI: 1.33–6.06; *p* = 0.007) and 3.53 (95% CI: 1.58–7.89; *p* = 0.002) in endometriosis patients, as compared to non-endometriosis women, respectively. However, the carcinogenic potential can be attributed to a series of occurrences. Ten hallmarks of cancer were purposed and included sustaining proliferative signaling, evading growth suppressors, avoiding immune destruction, enabling replicative immortality, tumor-promoting inflammation, activating invasion and metastasis, inducing or accessing vasculature, genome instability and mutation, resisting cell death, and deregulating cellular metabolism [4]. Tumor-promoting inflammation is an important hallmark among them. Inflammation has a central role in endometriosis pathogenesis, whereby it upregulates the production of chemokines, which are responsible for elevated macrophage accumulation and activation in ectopic endometrial deposits and peritoneal fluid [5]. Peritoneal fluid can further augment inflammation around abnormal endometrial stains by promoting reactive oxygen species production and lipid peroxidation [6]. This inflammation can induce endothelial dysfunction and even cause carcinogenesis [7].

In addition to endometriosis, pelvic inflammatory disease (PID) is another very common inflammatory disorder of female pelvic organs. PID is the inflammation of the female upper genital tract, involving the uterus, oviducts, ovaries, or pelvic peritoneum [8]. It clinically presents as pelvic organ tenderness, exhibited by cervical motion tenderness, adnexal tenderness, or uterine compression tenderness on pelvic examination. It has been reported that proinflammatory change, as caused by endometriosis or PID in this theme, can directly stimulate estrogen production [9] and probably activates eutopic endometrium and, thereafter, epithelial proliferation mainly through estrogen-driven inflammation [10]. Cytokines and chemokines then specifically attract leukocyte population and recruit effector cells that start the evolutionary process of inflammatory response [5], eventually leading to nitric oxide and oxygen radical production and subsequent DNA damage in the proliferating cells [11,12]. It has been suggested that tissues exposed to inflammatory milieu have an increased susceptibility to the carcinogenic process [13,14].

In 2016, the age-standardized incidence rate of Taiwanese women who suffered from cancer of the uterine corpus was reported to be 12.99 per 100,000 women subjects according to the annual registry report from Health Promotion Administration in the Ministry of Health and Welfare [15]. Uterine cancer is the most common gynecological cancer among Taiwanese female individuals. Most uterine corpus cancers are endometrial cancers, which originate from the uterine epithelium, while less than 2% are uterine sarcomas, which stem from the stromal tissues [16,17]. Previous results, investigating whether endometriosis or PID increases the risk of uterine cancer, were conflicting. A positive relationshiop between endometriosis and endometrial cancer has been revealed, but display a wide variation [18,19]. Among these studies, Kok et al. reported a high risk, four-fold increased odds risk [OR = 4.05, 95% confidence interval (95% CI) = 1.20 to 13.66] in 2266 endometriosis Taiwanese women with short claims data between 2003 and 2005 in a retrospective cohort study [20], whereas Rowlands et al. demonstrated a lowest OR of endometrial cancer 1.47 (95% CI = 1.00 to 2.17) in 128 endometriosis Australian women, which disappeared completely when the risk of uterine cancer in the first year after endometriosis diagnosis was excluded in a case-control study [19], suggesting a potential for increased detection among women with endometriosis, rather than a true association. Furthermore, Saraswart et al. also found no relationship between them in a retrospective cohort study [21]. With regard to the relationship between PID and uterine cancer, Yang et al. conducted a population-based retrospective cohort study and revealed an increased risk of endometrial cancer, whereas only PID patients who were followed for <2 years or were >50 years had a significant difference [22]. In contrast, Shen et al. also suggested no increased risk for uterine cancer in PID patients in a population-based retrospective cohort study [23]. It is very important to compare and delineate the associations among endometriosis and PID, as well as uterine corpus cancer, to determine which of these inflammatory diseases really leads to increased uterine cancer risk, which no studies have investigated thus far because these relationships may affect the positive treatment attitude towards these diseases to prevent from this most common gynecological cancer. Most carcinogens have an incubation period to develop malignant tumors, and the risk of time varying effect of the common gynecological inflammatory diseases, endometriosis, or PID on uterine cancer may change with time and may not be linear with time, therefore it is also import to investigate the risk of uterine cancer with time after diagnosis of these inflammatory diseases.

We hypothesized that the impacts of endometriosis and PID on uterine cancer are different, and the disease duration may have impacts, and a period of time is needed to result in the highest risk of uterine cancer. Time varying effect on uterine cancer development was estimated after endometriosis and PID diagnosis, as compared to the comparison controls on the same index date, which was defined as one year after endometriosis and PID diagnoses. To our knowledge, no study compares the risks of uterine cancer between patients with endometriosis and those with PID in different age groups. Furthermore, the relative risk (RR) of older women was inferred to be different from that of younger individuals.

## 2. Materials and Methods

### 2.1. Study Design and Data Source

This retrospective nationwide cohort study explored the absolute risk and RR of uterine corpus cancer in patients with endometriosis or PID compared with generally age-matched women. We collected data from the Longitudinal Generation Tracking Database 2000 (LGTD 2000). LGTD 2000 comprises two million individuals randomly sampled from the Taiwan National Health Insurance (NHI) Research Database (NHIRD). NHI was launched in 1995 and presently covers more than 99% of the Taiwanese population. LGTD 2000 is a representative subset data of NHIRD and has a similar distribution of demographics and health care utilization of the NHI data. In addition, the Taiwan Cancer Registry (TCR) datasets, a population-based cancer registry system confirmed by pathology reports and established in 1979, were used to collect the data for the diagnoses of malignant tumors, occurrence rate, the epidemiological data of patients, the date when the cancer was diagnosed, the site where the cancer occurred, histopathologic characteristics, staging, and the assessment for the control and prevention of malignant tumors. All these data were linked for the cross comparisons between LGTD 2000 and TCR by encrypted personal identification number. Thereafter, these research datasets were regulated by the Health and Welfare Data Science Center, Taiwan. The anonymous secondary information, from both LGTD 2000 and TCR, were applied in the research. To waive informed consent, approval was obtained from the Chung Shan Medical University Hospital Institutional Review Board (CSMUH No: CS17129).

### 2.2. Identification of Study Cohort and Exclusion Criteria

Initially, a total of 982,495 female individuals were involved in LGTD 2000. Diagnoses of endometriosis and PID were separately identified using code 617 for endometriosis (including adenomyosis as 617.0), and codes 614 and 615 for PID, and these codes were recruited based on the Ninth Edition form the Clinical Modification (ICD-9-CM). The patients, who had endometriosis or PID, were defined if related ICD-9-CM codes were associated with ≥2 outpatient visits or any hospitalization. In total, 202,776 patients were diagnosed as having endometriosis or PID between January 2000 and December 2014, and the other 779,719 individuals were never diagnosed as having endometriosis or PID during the study period. This study selected 135,236 patients with endometriosis or PID (20,510 endometriosis patients and 114,726 PID patients), as well as 135,236 age-matched women without endometriosis and PID as comparison cohort.

The index date was defined as one year after the first date of endometriosis or PID diagnosis. The lag period of one-year was taken into account for the surveillance bias arising from health care utilization related to the treatment of endometriosis or PID. When the diagnoses of endometriosis and PID increased the frequency of medical examinations within this one year, the probability of finding subclinical uterine corpus cancer also increased. Of the identified endometriosis or PID population, we excluded: (1) 49,922 female individuals who were diagnosed to have endometriosis or PID if these diagnoses were made before January 2002 or after December 2014 because of the possibility of the left-truncation [24] and right-censoring of data; (2) 3433 patients who were diagnosed to have any cancer (including uterine corpus cancer and Lynch syndrome) before the index date; (3) 2252 female beneficiaries aged <12 or >65 years on the index date; (4) 7783 female individuals with previous hysterectomy, oophorectomy, or salpingectomy; and (5) 4240 female individuals diagnosed to simultaneously have endometriosis and PID in the studied period. After exclusion of the females who have endometriosis or PID, the remaining females were regarded as comparisons and matched with endometriosis patients or PID patients individually, according to the age on the index date. All female individuals selected for the study were at risk on the index date. Finally, the study included 135,236 comparisons (general population without endometriosis and PID), 114,726 patients with PID, and 20,510 patients with endometriosis for evaluating the risk of uterine corpus cancer (full cohort). The flowchart for selection and classification of the study group is presented in Supplementary Figure S1.

### 2.3. Study Events and Covariates and Primary Outcomes

Occurrence of uterine corpus cancer was determined using the 1979–2014 TCR datasets. The diagnosis of malignant neoplasm of the uterine corpus was defined as ICD-9-CM code 182.x or ICD-10-CM code C54. Considering the influence on the subtype of uterine cancer, the ICD-O-3 morphology code was included from the TCR. The risks of uterine carcinoma (endometrial cancer) and sarcoma were evaluated for each study individual. We identified the following morphology codes as uterine sarcoma: 8800–8932, 8934–8941, 8959–8975, and 9141–9582 [25]. Moreover, comorbidities that the subjects had were defined according to IDC-9-CM codes, and then they were recruited into the study when they were diagnosed at least two times at outpatient visits or they were diagnosed once with the codes because of hospitalization in the baseline studied interval (during one year before the index date). The definitions of exposure, study event, and comorbidity are listed in Supplementary Table S1. The primary outcome was to compare the risk of uterine corpus cancer, including carcinoma and sarcoma, among patients with endometriosis and PID and comparisons. The secondary outcome was further to evaluate the RR of patients with endometriosis and PID at different age subgroups.

### 2.4. Propensity Score Matching

To decrease the unbalanced variables in patients with endometriosis and PID patients and comparison groups, propensity score matching (PSM) was defined and used as a sensitivity analysis for the adjustment of potential confounding effects. The propensity score was calculated as the odds of endometriosis according to the demographic data, including birth year, age on the index date, index year, marital status, education level, and comorbidities using logistic regression model. The comorbidities including obesity, renal disease, hypertension, diabetes mellitus, lipid dysfunction, cardiovascular disease, ischemic stroke, hyperthyroidism, hypothyroidism, chronic hepatitis, and chronic obstructive pulmonary disease were considered for performing the propensity score matching. We applied the nearest neighbor greedy algorithm to match the patients with PID and comparison individuals, the patients with endometriosis as a reference, using 1:1:1 ratio.

### 2.5. Sensitivity and Subgroup Analyses

Sensitivity analysis included conditional proportional hazard to evaluate the hazard ratios (HRs) of uterine cancer in the PSM population in addition to full cohort [26]. In addition, the sensitivity analysis included the subtype event risk evaluation of uterine carcinoma or uterine sarcoma. Hence, a sensitivity analysis was used to compare increased uterine cancer risk through stratified follow-up after the index date (0 to 24, 25 to 48, 49 to 72, 73 to 96, and 97 to 120 months). To observe the time varying effect of PID and endometriosis on uterine cancer risk, landmark analysis was performed [27]. Moreover, the analysis was stratified by the age subgroups on index date to find the possible interaction between age and endometriosis or PID, as well as to assess the RRs of uterine cancer at different age subgroups.

### 2.6. Statistical Analysis

The *p*-value of statistical hypothesis testing was less than 0.05 by two-tailed test of computing the statistical significance. We applied the maximum of standardized mean difference (Max SMD) to assess the balance of demographic and comorbidity variables in the research and defined the difference as more than 10% imbalance among these characteristics [28]. The rates of uterine cancer were assessed among endometriosis patients, PID patients, and comparison females. The subjects began to be followed since the index date, and the study was closed when uterine cancer happened or a death event occurred or until 31 December 2014, whenever the results occurred at that time. The period to follow the subject was defined as person-month. The incidence rate was then defined using per 100,000 person-year, and the 95% CIs were calculated by applying the Poisson regression mode. The Kaplan-Meier estimator was performed to calculate the cumulative incidence probability of uterine cancer among the study groups. The log-rank test was used to discriminate the differences among them. The HRs for the uterine corpus cancer in endometriosis or PID patients, using the comparison females as a reference, were defined. We used multiple Cox regressions to adjust the hazard ratio and determined the adjusted hazard ratio (aHR) for the occurrence of uterine cancer by the controlling for the demographic characteristics and comorbidities. We applied the conditional Cox regression model to define the aHR of endometriosis or PID patients in comparison with the comparison controls after PSM [29]. Significance level was determined to be *p* < 0.05. The SAS V.9.4 software (SAS Institute Inc., Cary, NC, USA) was permitted to apply.

## 3. Results

### 3.1. Baseline Demographics and Comorbidity Characteristics among Study Groups

Overall, we enrolled 135,236 age matched comparison controls, 114,726 PID patients, and 20,510 endometriosis patients to assess the uterine cancer risk, and endometriosis patients were found to be more centralized when they had a 1963 to 1975 birth year. More endometriosis patients exhibited that they had hypertension, lipid dysfunction, chronic hepatitis, and chronic obstructive pulmonary disease compared with the PID patients and comparison controls (Table 1). Thereafter, the HR of uterine corpus cancer for patients with endometriosis or PID, and the comparison cohort as a reference group, were calculated using multiple Cox regressions to estimate the aHR of uterine cancer after adjusting these covariates.

Using the odds of endometriosis as a reference, 20,478 subjects were assigned in each studied group after PSM was performed, and the demographic variables and comorbidities of them in each group were balanced well because the Max SMD of these characteristics was all less than 10% (Supplementary Table S2).

### 3.2. Uterine Corpus Cancer Risk Differences and Ratios among Study Groups

The medians of follow-up time were 9.1, 9.4, and 6.8 years in the comparison, PID, and endometriosis cohorts, respectively. During the whole observation period, the incidence proportion of uterine corpus cancer was 129 out of 135,236 (0.095%), 142 out of 114,726 (0.124%), and 61 out of 20,510 (0.297%) female individuals in the comparison, PID, and endometriosis cohorts, respectively. The incidence rates (per 100,000 person-years) of uterine cancer were 11.62 (95% CI, 9.78 to 13.81) in comparison, 14.62 (12.40 to 17.24) in PID, and 43.85 (34.12 to 56.36) in the endometriosis cohorts (Table 2). The medians of follow-up time were balanced to 6.8, 6.9, and 6.8 years in the comparison, PID, and endometriosis cohorts, respectively, after PSM (Table 3). Notably, the highest incidence rate, 43.54 (95% CI, 33.88 to 55.96), of uterine cancer was noted in the endometriosis group.

In Table 2, the absolute risk (rate difference), as well as RR (aHR) of uterine cancer, were estimated in the PID and endometriosis cohorts compared with the risk in the comparison cohort. For 100,000 person-years, the rate difference of uterine cancer incidence was 3.00 (95% CI = −0.13 to 6.13; *p* = 0.059) in PID cohort and 32.23 (95% CI = 21.04 to 43.41; *p* < 0.001) in the endometriosis cohort. Furthermore, the risk differences of endometrial cancer and uterine sarcoma were significantly increased as 28.64 (95% CI, 18.01 to 39.26) and 3.59 (95% CI = 0.11 to 7.08), respectively, in the endometriosis cohort. However, these significant risk differences were not observed in the PID cohort. After the covariates were adjusted, such as the year of index date, the age of index date, the marital condition, the level of education, and the comorbidities by applying the multiple Cox regression, the aHR of uterine cancer increased to a 1.30 times higher risk (95% CI = 1.02 to 1.65) in the PID and 2.94 (95% CI = 2.16 to 4.00) in the endometriosis cohorts compared to the comparison group. The aHRs of endometrial cancer and uterine sarcoma were significantly increased at 2.92-fold higher risk (95% CI = 2.12 to 4.03) and 5.83 (95% CI = 2.02 to 16.89), respectively, in the endometriosis cohort. The significantly increased aHRs were also not observed in the PID cohort. After PSM, the absolute risk differences of uterine cancer incidence were 1.33 (95% CI = −7.77 to 10.43) in the PID cohort and 29.17 (95% CI = 16.55 to 41.78) in the endometriosis cohort. The significantly increased risk was not observed in the PID (aHR = 1.12, 95% CI = 0.61 to 2.05), but it was found in the endometriosis (aHR = 2.83, 95% CI = 1.70 to 4.71) cohorts (Table 3). Although endometriosis patients had higher risk for the development of uterine corpus cancer, they had a greater proportion of hypertension, lipid dysfunction, chronic hepatitis, and chronic obstructive pulmonary disease compared to the PID patients and comparison controls in the full cohort, which were the direct result of being associated with obesity (Table 1). However, the hazard ratios of uterine cancer have been adjusted in the full cohort (Table 2) and the propensity score matching cohort (Table 3).

### 3.3. Cumulative Incidence of Uterine Cancer

**Figure 1:**
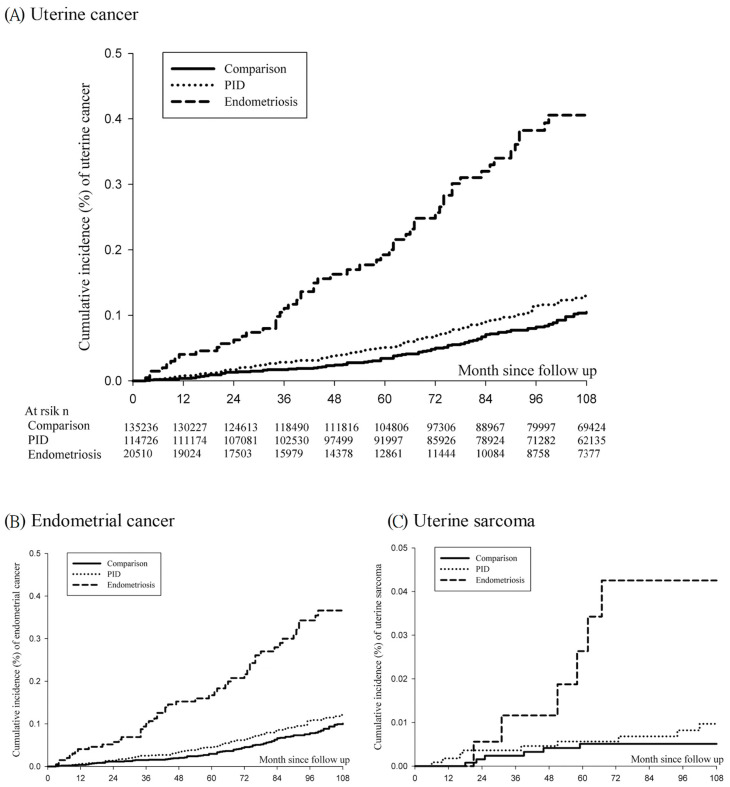
The cumulative incidence of uterine corpus cancer among endometriosis and pelvic inflammatory and comparison cohorts. (**A**) uterine cancer (log-rank *p* < 0.001); (**B**) endometrial cancer (log-rank *p* < 0.001); (**C**) uterine sarcoma (log-rank *p* < 0.001). PID, pelvic inflammatory disease.

Endometriosis patients had higher cumulative incidence rates in comparison with PID patients and the comparison controls [Figure 1, log-rank *p* < 0.001]. Figure 1A indicates the cumulative incidence rates of uterine cancer. In the comparison cohort, the cumulative incidence rates were 0.013%, 0.024%, 0.050%, and 0.083% at 24, 48, 72, and 96 months after index date, respectively. In the PID cohort, the cumulative incidence rates were 0.017%, 0.039%, 0.069%, and 0.115%, respectively. In the endometriosis cohort, the cumulative incidence rates were 0.063%, 0.163%, 0.257%, and 0.394% at 24, 48, 72, and 96 months, respectively. Figure 1B,C reveal that the endometriosis cohort had significantly higher cumulative incidence rates of endometrial cancer and uterine sarcoma (log-rank *p* < 0.001 and log-rank *p* < 0.001, respectively). In addition, similar results of higher cumulative incidence rates of uterine cancer and endometrial cancer were observed in the endometriosis cohort after PSM (Supplementary Figure S2, log-rank *p* < 0.001 and log-rank *p* < 0.001).

### 3.4. Sensitivity and Subgroup Analyses

The sensitivity analyses revealed similar results in full cohort (Table 2) and the PSM population (Table 3). Patients with endometriosis were more susceptible to uterine cancer than the comparisons and those with PID in full cohort and PSM population. In addition, patients with endometriosis still had a higher risk of endometrial cancer after exclusion of those with uterine sarcoma (Table 2).

**Figure 2:**
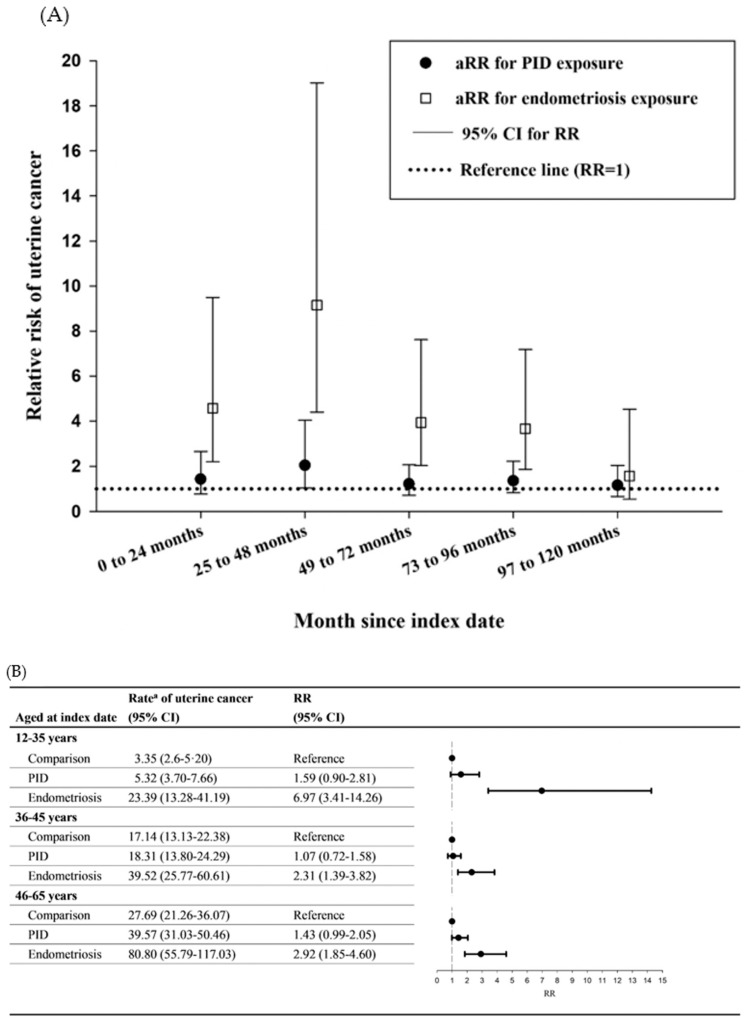
Age-dependent and time-varying effect of endometriosis on uterine corpus cancer. (**A**) Subgroup analysis for uterine corpus cancer among age and birth year matched population. (**B**) Landmark analysis for time varying effect on the risk of uterine corpus cancer. ^a^ Crude rate, per 100,000 person-years. PID, pelvic inflammatory disease; RR, relative risk; 95% CI, 95% confidence interval. aRR, adjusted relative risk.

The landmark analysis was performed to observe the time varying effect of PID or endometriosis on the risk of uterine corpus cancer. It revealed the highest RR estimated during 25 to 48 months after the index date (converted as during 37 to 60 months after PID or endometriosis diagnosis) in the PID [adjusted relative risk (aRR) = 2.04, 95% CI = 1.04 to 4.04] and endometriosis (aRR = 9.15, 95% CI = 4.40 to 19.02) cohorts (Figure 2A and Table 4).

Figure 2B showed the risk of uterine corpus cancer stratified by the age subgroups on index date. The rate of uterine cancer increased with older female subgroups. However, the RR was only significantly increased in endometriosis cohort, but not in the PID cohort. After age subgroup analysis, it showed that younger female individuals (aged 12 to 35 years) had the highest RR in the endometriosis cohort (RR = 6.97, 95% CI = 3.41 to 14.26), and older subjects (46 to 65 years) did not exhibit higher RR (RR = 2.92, 95% CI = 1.85 to 4.60) than the younger subgroup. In the PID cohort, different age subgroups did not have different RR for uterine cancer. Therefore, a significant interaction effect between age and endometriosis was observed.

## 4. Discussion

With a large sample size and extended follow-up, the present study provided evidence that endometriosis may be an independent risk factor for not only endometrial cancer, but also uterine sarcoma. First, endometriosis influenced the risk of new-onset uterine cancer more than did PID. Second, endometriosis exerted the most significantly increased uterine cancer risk in patients with endometriosis for 37 to 60 months. Third, younger females aged 12 to 35 years with endometriosis were at the greatest increased risk of uterine cancer, as compared with those of other age subgroups. Fourth, patients with endometriosis are more susceptible to not only endometrial cancer but uterine sarcoma. The difference between the impacts of exposure of exposure to endometriosis and PID on uterine cancer accounted for those patients with endometriosis who became more prone to develop uterine cancer, including endometrial cancer and uterine sarcoma. Epidemiological studies have demonstrated a contradictory relationship between endometriosis and endometrial cancer risk. A large retrospective cohort study that enrolled 45,790 Danish female individuals with endometriosis performed univariate analysis to reveal an elevated risk of endometrial cancer with standardized incidence ratio (SIR) 1.43 (95% CI = 1.13 to 1.79) after endometriosis was diagnosed at > 1 year, as well as SIR 1.51 (95% CI = 1.15 to 1.95) ≥ 10 years [18]. By contrast, in a prospective cohort study among nurses’ health study II (NHSII) participants, no association was observed between endometrial cancer and endometriosis [30]. However, in the NHSII study, the population was restricted to USA nurses, and the participants were not surgically evaluated for endometriosis. Moreover, Melin et al. used the National Swedish Inpatient Register and linked the data to the National Swedish Cancer Register to associate endometrial cancer with 65,349 women with a diagnosis coded for endometriosis, and could not find an increased risk of endometrial cancer (SIR = 1.14, 95% CI = 093 to 1.39) [31]. Furthermore, we collected the recent eight studies in a review article and estimated the combined odds ratio for endometrial cancer as 1.60 (95% CI = 1.10 to 2.34) in women with endometriosis, which supports our current findings [32].

The inflammation caused by endometriosis is presumed to be a cause that is related to the occurrence of uterine cancer. Inflammatory mediators, such as tumor necrosis factor-α (TNF-α) and interleukin-1β, can increase human endometrial haptoglobin production in female individuals with endometriosis [33]. One group of the nuclear factor-κB (NF-κB) activators are the proinflammatory cytokines, including TNF-α and interleukin-1β [34]. Aberrant NF-κB expression may activate malignancy-promoting signaling pathways in both cancer cells and cancer-associated inflammatory cells [9,14,35]. Estrogen dominance in endometriosis and its downstream signal targets were demonstrated to have a paramount role in disease development and maintenance, with their actions on eutopic endometrium and epithelial proliferation, mainly through estrogen-driven inflammation and progesterone resistance [10,36]. Estrogen promotes epithelial proliferation throughout the reproductive organs and may drive proliferative diseases, such as endometriosis and endometrial cancer [37,38]. Both endometriosis and endometrial cancer are hormone-related diseases, with an increased risk in female individuals exposed to increased estrogen levels [39]. Hence, inflammation may act in tandem with estrogen to cause endometrial cancer. Epidemiological, biological, and molecular data all indicate a probable correlation between these two diseases [13,39]. Moreover, evidence from clinical studies revealed that CTLA4-based autoimmunity is related to the maintenance of chronic inflammation in the peritoneal environment, probably resulting from endometriosis-related peritoneal fluid [5,40].

Women with PID have been demonstrated to exhibit an increased uterine cancer risk by a population-based retrospective cohort study, but only PID patients who were followed for <2 years displayed significant difference (aHR = 7.91, 95% CI = 2.92 to 21.4) [22]. Moreover, only 18 cancer events were found in the <2 year follow-up subgroup. Interestingly, the significant risk only occurred in PID women age >50 years (aHR = 2.45, 95% CI = 1.29 to 4.65), and only 21 cancer events were found in this subgroup. However, there have been few reports of a significant association between PID and uterine cancer. The SIR of uterine/endometrial cancer < 1 year after PID diagnosis was suggested to be 20.1 (95% CI = 16.3 to 24.4), but the SIR of uterine/endometrial cancer > 1 year after PID diagnosis was reduced to 0.9 (95% CI = 0.8 to 1.0) [41]. In our study, endometriosis was noted to influence uterine cancer occurrence more than PID. This finding could be partially explained by the fact that patients with PID were probably cured, with the inflammation not lasting as long as that in endometriosis. Moreover, it may partially be attributable to the fact that patients with PID had a higher outpatient diagnosis rate followed by decreased inflammation severity than did those with endometriosis.

To our knowledge, this is the first nationwide population study to explore endometriosis as an independent risk factor for uterine sarcoma. It has been reported that uterine sarcoma displays a variable rate of estrogen receptor (ER) [42]. ER was detected in 53% of low-grade endometrial stromal sarcoma (ESS), 45% of uterine leiomyosarcoma (uLMS), (the most common subtype among uterine sarcomas), 23% of high-grade ESS, and 47% of all uterine sarcomas. National Comprehensive Cancer Network Guidelines Version 1.2021 for uterine sarcoma has recommended anti-estrogen hormone therapy for low-grade ESS or ER receptor-positive uLMS. ER levels may be elevated in the secretory phase endometrium of endometriosis females mainly with inflammatory change compared to controls, which may result in estrogen dominance and increase estrogenic activity, harmful inflammation, and cell proliferation [10,36,43]. Proinflammatory change can further stimulate estrogen production [9] and is inferred to increase ligand with ERs of not only endometrium, but also stromal tissues and then promotes cell proliferation. However, PID is mainly related to microbial infection but not directly related to estrogen expression. The correlation between endometriosis and uterine sarcoma related to the estrogen and ER seems to be more relevant than the correlation between PID and uterine sarcoma. This implies that endometrial cancer and uterine sarcoma are not only related to inflammation but also to estrogen. Therefore, a notable finding in this study is that patients with endometriosis have an increased HR for uterine sarcoma. In addition, the AXL receptor tyrosine kinase gene was reported to be dysregulated in endometriosis and cancers associated with the endometrium and myometrium [44,45]. Moreover, endometrial stromal sarcoma, a common uterine sarcoma subtype, may be associated with endometriosis because it was seen arising from endometriosis lesions or foci, as per sporadic case reports [46,47].

In this study, the highest RR was estimated from 25 to 48 months after the index date (about three to five years after diagnosis) in the endometriosis cohort. Furthermore, a significant interaction effect was observed between age and endometriosis, which implied that different age subgroups had different rates of endometriosis. Although the rate of uterine cancer increased with older female subgroups, younger female individuals (aged 12 to 35 years) with endometriosis had the highest RR of uterine cancer after age stratification. This may result from lower incidence rate of uterine cancer in comparisons and higher incidence of endometriosis in this age subgroup. The fertile capacity in Taiwanese women is reduced if they are older than 35 years. In addition, the age of menopause for women is between 45 and 55. Therefore, the age subgroups were defined as 12–35 years, 36–45 years, and 46–65 years. The age subgroup 46–65 years was defined menopause status. The relative risks of endometriosis patients for uterine corpus cancer were highest (RR: 6.97, 95% CI:3.41–14.26) in patients aged 12–35 years in comparison with their normal comparisons among the age subgroups. The highest estrogen levels were inferred in this age subgroup. Nevertheless, this aspect should be further explored in the future from another perspective with regard to whether younger female individuals, who generally have a lower uterine cancer risk, develop the cancer in a endometriosis-related fashion. Notably, endometriosis may be a prominently crucial risk factor for uterine cancer early in young Taiwanese female individuals.

The current study has some strengths. A nationwide larger cohort, which covered more than 99% of the total Taiwan female population, was recruited from a sufficiently long period (2000 to 2014) datasets, enabling authors to track the subsequent risk of uterine cancers in the patients with endometriosis, those with PID, and controls. This might minimize the possibility of recall bias or biased follow-up and biased results because of the left-truncation and right-censoring information [48], mainly when the short time-window is used [49]. The diagnosis of uterine cancer was confirmed by the cross-link between both LGTD 2000 and TCR for the large-scale study population to lower the selection bias. Our study adjusted several possible confounding factors and performed PSM, which further validated the study results. Furthermore, to manage the confounding bias arising from increased health care utilization within one year after diagnosis of PID or endometriosis, the index date was lagged by one year. In addition, the sensitivity analyses were performed to verify our novel results. Overall, given the high disease burden of cancers, implications of the present include the establishment of screening and education programs for populations at risk of uterine cancer.

The study also has some limitations. Only a few cases of uterine sarcoma could be identified for statistical analysis after PSM because of low incidence of the disease itself. Endometrioid endometrial cancer constitutes the majority of endometrial cancer cases (more than 80%), which are generally estrogen-dependent tumors [50]. By contrast, serous endometrial cancer (SEC) and clear cell endometrial cancer (CCEC) only account for approximating 10% and around 3% (~10% and 3%), respectively. Only a small number of cases of SEC and CCEC could be subdivided from endometrial cancer for classification analysis. Considering the relationships of endometriosis and endometrial cancer was previously explained; however, we believe that the risk of endometrioid endometrial cancer will be further raised after exclusion of SEC and CCEC. Another limitation was that the menopausal status was not included as a variable for analysis. Therefore, subgroup analysis for age was applied to analyze the menopausal status. Women aged > 45 years, who were menopausal or close to menopause, were included in the age stratification analysis. Although the rate of uterine cancer increased with older female subgroups in patients with PID or endometriosis, only younger female individuals (aged 12 to 35 years) with endometriosis still had the highest increased RR of uterine cancer. Other limitations included no known information—whether the considered diagnosis of endometriosis was clinical or surgical and whether the diagnosis of PID was clinical or whether there was microbiological confirmation. The use of the nearest neighbor greedy algorithm might interfere with the interpretation of the results. The selected “healthy” individuals who were probably very similar to each other and distinct from individuals with endometriosis. In this case, it reduces the variability of the “normal” population, and this can maximize differences between study groups. However, in our primary analysis, there were 135,236 patients with endometriosis or PID (20,510 patients with endometriosis, and 114,726 patients with PID), and they were individual age-matched with 135,236 comparisons (general population without endometriosis and PID) who were at risk on the index date. We selected the 135,236 comparisons as “normal” group, and the crude and adjusted risks of endometrial cancer were compared with endometriosis patients and PID patients. In addition, we performed the propensity score analysis to validate the primary finding. A surveillance bias might exist, but nevertheless, it was managed by lagging the index date by one year after PID or endometriosis diagnosis [41]. Our current findings may be explained by that that younger females having uterine cancer may be really related to endometriosis, which occurred more frequent in this age subgroup. Similarly, Mogensen et al. revealed that younger female individuals (aged 30–39 years) with endometriosis have the highest SIR in Denmark [18]. However, the left-truncation data still confounded our results because we noted a lower RR in the elderly population. Therefore, the RR might have been underestimated in elderly individuals.

## 5. Conclusions

Younger females with endometriosis and patients who had endometriosis for three to five years were at greatest risk of uterine cancer, as compared with propensity score-matched populations and compared with patients with PID. Clinical implications of the present study suggested screening and early treatments for the identified endometriosis populations susceptible to uterine corpus cancer. We especially recommend that the younger females, who had endometriosis, should actively follow the endometriosis and receive treatment. In addition, it is very important and worthy to stress the necessity for the performance of combined personalized treatment, taking into consideration both psychological and physical issues of the endometriosis because of the diverse symptoms and signs, especially considering mental health aspects [51].

## Figures and Tables

**Table 1:** Characteristics of exposure (pelvic inflammatory disease or endometriosis) cohort and age matched comparison cohort.

	No. (%)			
Characteristics	Comparison	PID	Endometriosis	Max SMD
**No. in cohort**	135,236	114,726	20,510	
**Birth year**				32.95%
1937–1949	3474 (2.57%)	3274 (2.85%)	200 (0.98%)	
1950–1962	29,272 (21.65%)	23,653 (20.62%)	5619 (27.40%)	
1963–1975	49,587 (36.67%)	40,605 (35.39%)	8982 (43.79%)	
1976–1988	46,103 (34.09%)	41,117 (35.84%)	4986 (24.31%)	
1989–2002	6800 (5.03%)	6077 (5.30%)	723 (3.53%)	
**Year of index**				45.67%
2002–2006	80,807 (59.75%)	71,948 (62.71%)	8859 (43.19%)	
2007–2010	31,435 (23.24%)	25,854 (22.54%)	5581 (27.21%)	
2011–2014	22,994 (17.00%)	16,924 (14.75%)	6070 (29.60%)	
**Age on index date**				50.14%
12–25	29,606 (21.89%)	27,304 (23.8%)	2302 (11.22%)	
26–35	43,757 (32.36%)	38,537 (33.59%)	5220 (25.45%)	
36–45	36,730 (27.16%)	29,119 (25.38%)	7611 (37.11%)	
46–55	21,304 (15.75%)	16,168 (14.09%)	5136 (25.04%)	
56–65	3839 (2.84%)	3598 (3.14%)	241 (1.18%)	
**Marital status**				21.24%
Single	59,712 (44.15%)	39,212 (34.18%)	7096 (34.60%)	
Married	65,558 (48.48%)	63,066 (54.97%)	11,873 (57.89%)	
Others	9966 (7.37%)	12,448 (10.85%)	1541 (7.51%)	
**Education level (years)**				27.58%
<7	25,079 (18.54%)	23,337 (20.34%)	2792 (13.61%)	
7–9	25,627 (18.95%)	25,655 (22.36%)	3697 (18.03%)	
10–12	52,137 (38.55%)	47,233 (41.17%)	8323 (40.58%)	
≥13	32,393 (23.95%)	18,501 (16.13%)	5698 (27.78%)	
**Comorbidities**				
Obesity	489 (0.36%)	499 (0.43%)	145 (0.71%)	4.74%
Renal disease	1036 (0.77%)	1303 (1.14%)	298 (1.45%)	6.56%
Hypertension	5037 (3.72%)	5016 (4.37%)	1359 (6.63%)	13.13%
Diabetes mellitus	2682 (1.98%)	2959 (2.58%)	712 (3.47%)	9.15%
Lipid dysfunction	4183 (3.09%)	4749 (4.14%)	1273 (6.21%)	14.83%
CVD	1317 (0.97%)	1912 (1.67%	409 (1.99%)	8.45%
Ischemic stroke	358 (0.26%)	369 (0.32%)	105 (0.51%)	3.98%
Hyperthyroidism	1591 (1.18%)	1925 (1.68%)	431 (2.10%)	7.29%
Hypothyroidism	511 (0.38%)	597 (0.52%)	227 (1.11%)	8.50%
Chronic hepatitis	4200 (3.11%)	5528 (4.82%)	1361 (6.64%)	16.46%
COPD	3949 (2.92%)	4651 (4.05%)	1034 (5.04%)	10.87%

PID, pelvic inflammatory disease; CVD, cardiovascular disease; COPD, chronic obstructive pulmonary disease; Max SMD, maximum of standardized mean difference, the unbalanced characteristic was observed with the Max SMD > 10%.

**Table 2:** Incidence rate of uterine corpus cancer between exposure (pelvic inflammatory disease or endometriosis) and comparison cohorts.

	Comparison	PID	Endometriosis
**No. in cohort**	135,236	11,4726	20,510
**Follow up person-years**	1,109,867	971,163.2	139,105.3
**Median of follow up year**	9.1	9.4	6.8
**Uterine corpus cancer**			
Event	129	142	61
Rate ^a^ (95% CI)	11.62 (9.78 to 13.81)	14.62 (12.40 to 17.24)	43.85 (34.12 to 56.36)
Risk difference ^b^ (95% CI)	Reference	3.00 (−0.13 to 6.13)	32.23 (21.04 to 43.41)
aHR ^c^ (95% CI)	Reference	1.30 (1.02 to 1.65)	2.94 (2.16 to 4.00)
**Uterine cancer—endometrial cancer**			
Event	121	133	55
Rate ^a^ (95% CI)	10.90 (9.12 to 13.03)	13.69 (11.55 to 16.23)	39.54 (30.36 to 51.50)
Risk difference ^b^ (95% CI)	Reference	2.79 (−0.24 to 5.82)	28.64 (18.01 to 39.2)
aHR ^c^ (95% CI)	Reference	1.28 (1.00 to 1.64)	2.92 (2.12 to 4.03)
**Uterine cancer—sarcoma**			
Event	8	9	6
Rate ^a^ (95% CI)	0.72 (0.36 to 1.44)	0.93 (0.48 to 1.78)	4.31 (1.94 to 9.60)
Risk difference ^b^ (95% CI)	Reference	0.21 (−0.58 to 0.99)	3.59 (0.11 to 7.08)
aHR ^c^ (95% CI)	Reference	1.21 (0.46 to 3.14)	5.83 (2.02 to 16.89)

^a^ Crude rate, per 100,000 person-years ^b^ Difference of crude rate, per 100,000 person-years ^c^ aHR: adjusted hazard ratio, multiple Cox regression is conducted to adjust the covariates including year of index, age on index date, marital status, education level, and comorbidities. PID, pelvic inflammatory disease; 95% CI, 95% confidence interval.

**Table 3:** Incidence rate of uterine corpus cancer among study groups after propensity score matching.

	Comparison	PID	Endometriosis
**No. in cohort**	20,478	20,478	20,478
**Follow up person-years**	139,142.4	140,099.2	139,049.3
**Median of follow up (year)**	6.8	6.9	6.8
**Uterine corpus cancer**			
Event	20	22	61
Rate ^a^ (95% CI)	14.37 (9·27 to 22.28)	15.70 (10.34 to 23.85)	43.54 (33.88 to 55.96)
Risk difference ^b^ (95% CI)	Reference	1.33 (−7.77 to 10.43)	29.17 (16.55 to 41.78)
aHR ^c^ (95% CI)	Reference	1.12 (0.61 to 2.05)	2.83 (1.70 to 4.71)

^a^ Crude rate, per 100,000 person-years. ^b^ Difference of crude rate, per 100,000 person-years. ^c^ aHR: adjusted hazard ratio estimated by the conditional Cox regression model (stratified by paired group). PID, pelvic inflammatory disease; 95% CI, 95% confidence interval.

**Table 4:** Landmark analysis for time varying effect on the risk of uterine corpus cancer.

Landmark Interval	Comparison	PID	Endometriosis
**0 to 24 months**			
Follow up person-years	259,763.8	221,801.5	37,896.33
Event	18	22	12
Rate ^a^ (95% CI)	6.93 (4.37 to 11.00)	9.92 (6.53 to 15.06)	31.67 (17.98 to 55.76)
aRR (95% CI)	Reference	1.43 (0.77 to 2.67)	4.57 (2.20 to 9.49)
**25 to 48 months**			
Follow up person-years	236,147.6	204,416.3	31,771.5
Event	13	23	16
Rate ^a^ (95% CI)	5.50 (3.20 to 9.48)	11.25 (7.48 to 16.93)	50.36 (30.85 to 82.21)
aRR (95% CI)	Reference	2.04 (1.04 to 4.04)	9.15 (4.40 to 19.02)
**49–72 months**			
Follow up person-years	199,574	175,221.2	24,458.83
Event	27	29	13
Rate ^a^ (95% CI)	13.53 (9.28 to 19.73)	16.55 (11.50 to 23.82)	53.15 (30.86 to 91.54)
aRR (95% CI)	Reference	1.22 (0.72 to 2.07)	3.93 (2.03 to 7.61)
**73–96 months**			
Follow up person-years	168,977.7	149,962.8	19,092.83
Event	29	35	12
Rate ^a^ (95% CI)	17.16 (11.93 to 24.70)	23.34 (16.76 to 32.50)	62.85 (35.69 to 110.67)
aRR (95% CI)	Reference	1.36 (0.83 to 2.23)	3.66 (1.87 to 7.18)
**97–120 months**			
Follow up person-years	130,205	116,527.8	13,802.42
Event	24	25	4
Rate ^a^ (95% CI)	18.43 (12.36 to 27.50)	21.45 (14.50 to 31.75)	28.98 (10.88 to 77.22)
aRR (95% CI)	Reference	1.16 (0.67 to 2.04)	1.57 (0.55 to 4.53)

^a^ Crude rate, per 100,000 person-years. PID, pelvic inflammatory disease; 95% CI, 95% confidence interval; aRR, adjusted relative risk.

## Data Availability

Datasets from The Longitudinal Generation Tracking Database 2000 and Taiwan Cancer Registry are available through a request to the Health and Welfare Data Science Center (HWDSC) [52]. However, the data are not publicly available due to their containing information that could compromise the privacy of research participants.

## Supplementary Materials

The following supporting information can be downloaded at: https://www.mdpi.com/article/10.3390/cancers15030833/s1. Table S1. The definitions of exposure and study event and comorbidity in the study. Table S2. Baseline characteristics among study groups after propensity score matching. Figure S1. The detailed enrolled flow chart for uterine corpus cancer among pelvic inflammatory disease, endometriosis, and control cohorts. ^a^ The index date was defined as one year after the first date of diagnosis with endometriosis or PID. LGTD 2000, The Longitudinal Generation Tracking Database 2000; PID, pelvic inflammatory disease; PSM, propensity score matching. Figure S2. The cumulative incidence of uterine corpus cancer among endometriosis and pelvic inflammatory disease and comparison cohorts after propensity score matching. (A) uterine cancer (log-rank *p* < 0.001); (B) endometrial cancer (log-rank *p* < 0.001). The cumulative incidence of uterine sarcoma has not been provided since the few cases of uterine sarcoma were obtained in database. PID, pelvic inflammatory disease.
