# Supplementary material for: The Risk of Endometrial Cancer and Uterine Sarcoma Following Endometriosis or Pelvic Inflammatory Disease
Source: Cancers (Basel). 2023 Jan 29;15(3):833. doi: 10.3390/cancers15030833 (PMC9913480; doi:10.3390/cancers15030833)
Supplement: Supplementary file 1 [file cancers-15-00833-s001.zip › cancers-2067118-supplementary.pdf]

Supplementary Information

Supplementary Table S1. The definitions of exposure and study event and comorbidity in the study

Variables	ICD-9-CM codes identified from LGTD 2000
**Exposures**	
Endometriosis	617.x
PID	614.x and 615.x
**Study Events**	
Malignant neoplasm of uterine corpus	The ICD-9 codes 182.x, or ICD-10 codes C54 identified from Taiwan Cancer Registry datasets
**Comorbidities**	
Obesity	278.x
Renal disease	580.x-589.x
Hypertension	401.x-405.x
Diabetics mellitus	250.x
Lipid dysfunction	272.x
CVD	410.x-414.x
Ischemic stroke	433.x-436.x
Hyperthyroidism	242.x
Hypothyroidism	244.x
Chronic hepatitis	571.4, 571.5, 571.6, 571.7, 571.8, 571.9, 573.1, 573.2, 573.3
COPD	491.x, 492.x, 496.x

PID, pelvic inflammatory disease; CVD, cardiovascular disease; COPD, chronic obstructive pulmonary disease ; ICD-9-CM, International Classification of Diseases, 9th Revision, Clinical Modification; LGTD 2000, Longitudinal Generation Tracking Database 2000.

Supplementary Table S2. Baseline characteristics among study groups after propensity score matching.

	No. (%)			
**Characteristics**	**Comparison**	**PID**	**Endometriosis**	**Max SMD**
**No. in cohort**	20478	20478	20478	
**Birth year**				8.56%
1937-1949	251 (1.23%)	259 (1.26%)	200 (0.98%)	
1950-1962	5515 (26.93%)	5448 (26.60%)	5614 (27.41%)	
1963-1975	8838 (43.16%)	8528 (41.64%)	8957 (43.74%)	
1976-1988	5148 (25.14%)	5511 (26.91%)	4984 (24.34%)	
1989-2002	726 (3.55%)	732 (3.57%)	723 (3.53%)	
**Year of index**				2.39%
2002-2006	8857 (43.25%)	8928 (43.60%)	8859 (43.26%)	
2007-2010	5598 (27.34%)	5574 (27.22%)	5578 (27.24%)	
2011-2014	6023 (29.41%)	5976 (29.18%)	6041 (29.50%)	
**Age at index date**				0.00%
12-25	2297 (11.22%)	2295 (11.21%)	2302 (11.24%)	
26-35	5206 (25.42%)	5202 (25.40%)	5220 (25.49%)	
36-45	7537 (36.81%)	7663 (37.42%)	7598 (37.10%)	
46-55	5201 (25.40%)	5091 (24.86%)	5117 (24.99%)	
≥ 56	237 (1.16%)	227 (1.11%)	241 (1.18%)	
**Marital status**				4.03%
Single	7000 (34.18%)	7145 (34.89%)	7076 (34.55%)	
Married	11962 (58.41%)	11786 (57.55%)	11861(57.92%)	
Others	1516 (7.40%)	1547 (7.55%)	1541 (7.53%)	
**Education level (years)**				2.47%
< 7	2861 (13.97%)	2812 (13.73%)	2792 (13.63%)	
7-9	3651 (17.83%)	3666 (17.90%)	3695 (18.04%)	
10-12	8282 (40.44%)	8281 (40.44%)	8319 (40.62%)	
≥ 13	5684 (27.76%)	5719 (27.93%)	5672 (27.70%)	
**Comorbidities**				
Obesity	141 (0.69%)	125 (0.61%)	144 (0.70%)	1.15%
Renal disease	259 (1.26%)	278 (1.36%)	297 (1.45%)	1.60%
Hypertension	1408 (6.88%)	1336 (6.52%)	1353 (6.61%)	0.34%
Diabetes mellitus	689 (3.36%)	632 (3.09%)	708 (3.46%)	2.09%
Lipid dysfunction	1288 (6.29%)	1175 (5.74%)	1264 (6.17%)	1.84%
CVD	394 (1.92%)	374 (1.83%)	405 (1.98%)	1.11%
Ischemic stroke	95 (0.46%)	103 (0.50%)	102 (0.50%)	0.56%
Hyperthyroidism	412 (2.01%)	418 (2.04%)	430 (2.10%)	0.62%
Hypothyroidism	206 (1.01%)	185 (0.90%)	222 (1.08%)	1.82%
Chronic hepatitis	1371 (6.69%)	1313 (6.41%)	1350 (6.59%)	0.73%
COPD	1050 (5.13%)	1032 (5.04%)	1032 (5.04%)	0.00%

PID, pelvic inflammatory disease; CVD, cardiovascular disease; COPD, chronic obstructive pulmonary disease ; Max SMD, maximum of standardized mean difference, the balanced characteristic was observed with the Max SMD<10%.

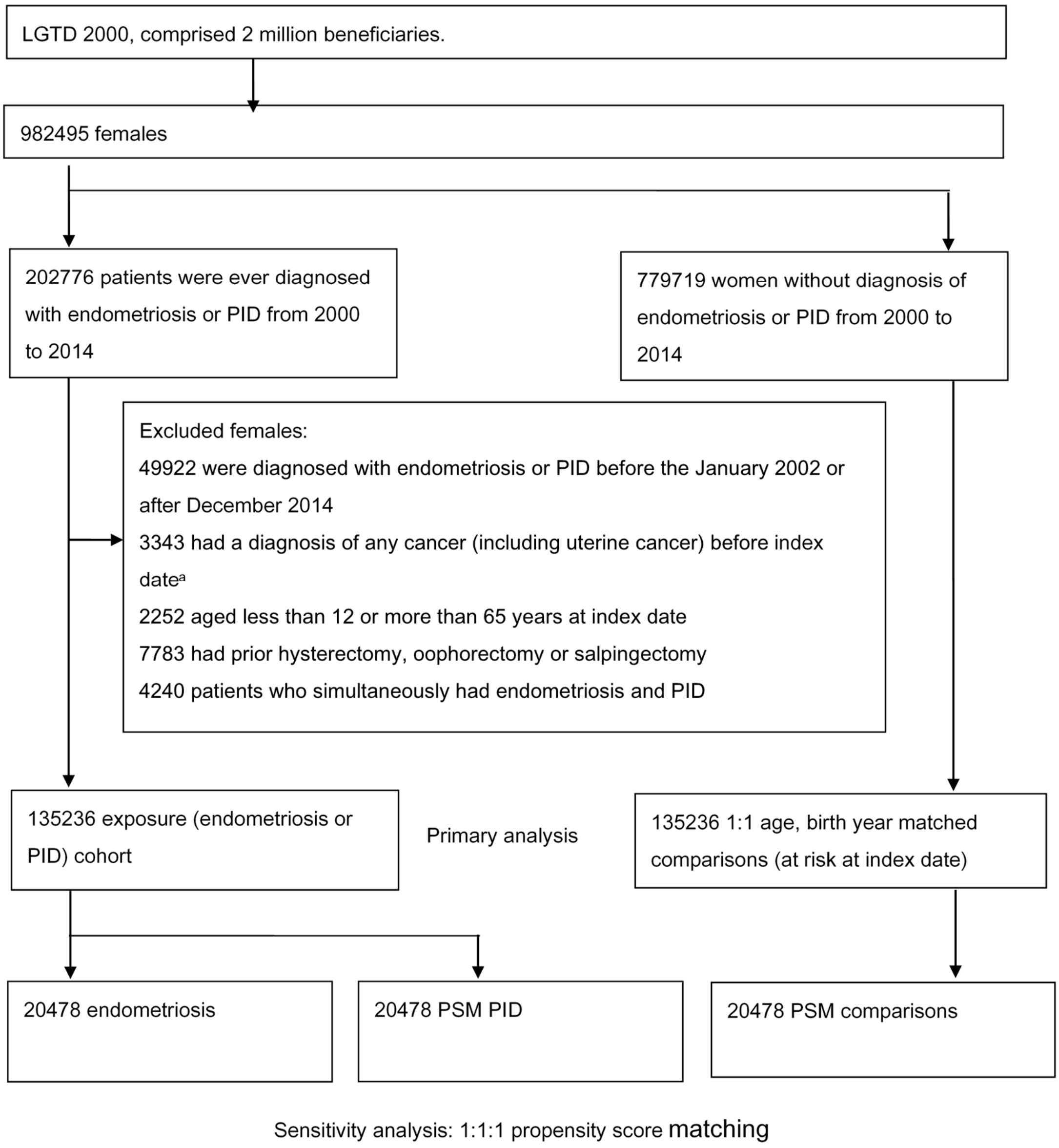

Supplementary Figure S1. The detailed enrolled flow chart for uterine corpus cancer among pelvic inflammatory disease, endometriosis and control cohorts.
[a] The index date was defined as one year after the first date of diagnosis with endometriosis or PID. LGTD 2000, The Longitudinal Generation Tracking Database 2000; PID, pelvic inflammatory disease; PSM, propensity score matching.

(A) Uterine cancer

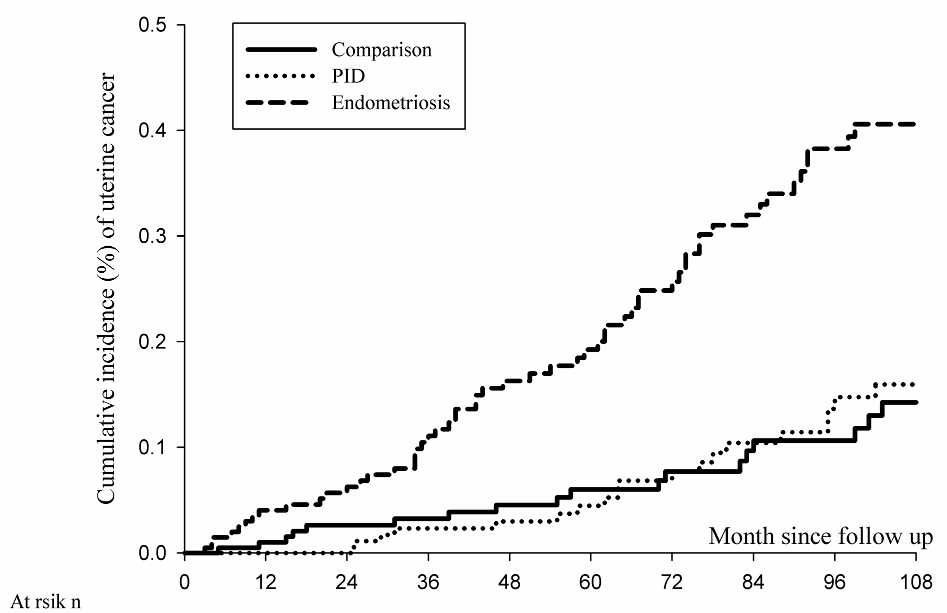

At rsik n										
Comparison	20478	18995	17476	15968	14382	12838	11411	10066	8763	7368
PID	20478	19064	17553	16040	14459	12939	11518	10163	8846	7458
Endometriosis	20478	19005	17489	15974	14375	12858	11444	10084	8758	7377

(B) Endometrial cancer

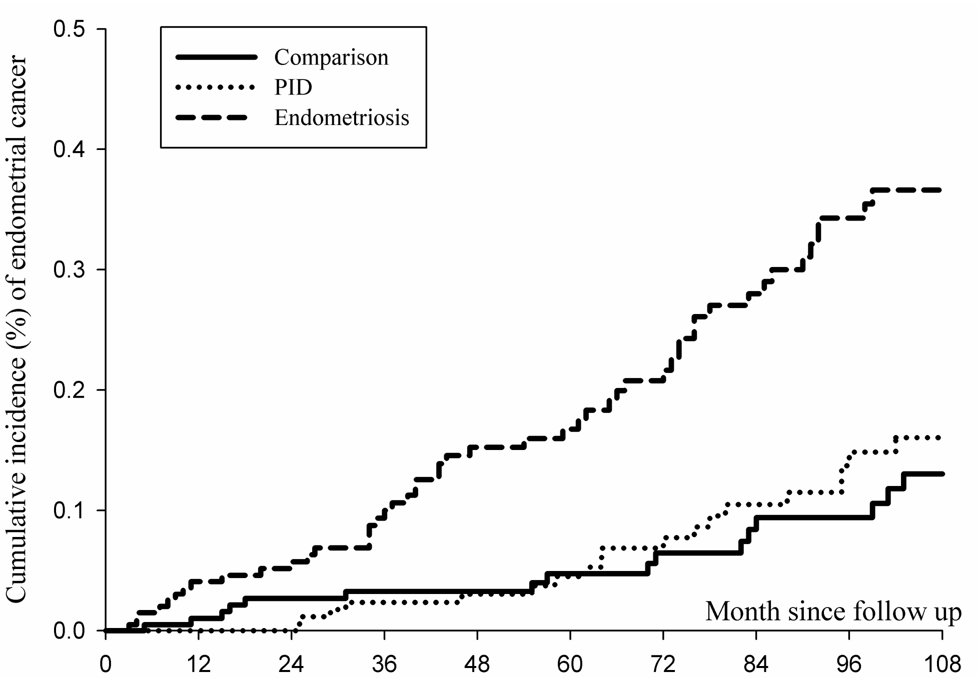

Supplementary Figure S2. The cumulative incidence of uterine corpus cancer among endometriosis and pelvic inflammatory disease and comparison cohorts after propensity score matching.

(A) uterine cancer (log-rank *P*< .001); (B) endometrial cancer (log-rank *P*< .001)· The cumulative incidence of uterine sarcoma has not been provided since the few cases of uterine sarcoma were obtained in database. PID, pelvic inflammatory disease.
